# Evaluation tool for a gastroenterostomy simulated training^1^

**DOI:** 10.1590/s0102-865020190030000008

**Published:** 2019-03-18

**Authors:** Márcio Alencar Barreira, Delano Gurgel Siveira, Hermano Alexandre Lima Rocha, Luiz Gonzaga de Moura, Charles Jean Gomes de Mesquita, Gleydson Cesar de Oliveira Borges

**Affiliations:** I MD, Surgical Oncologist, Hospital Universitário Walter Cantídio, Universidade Federal do Ceará (UFC), Fortaleza-CE, Brazil. Conception and design of the study; technical procedures; acquisition, interpretation and analysis of data; manuscript preparation and writing.; II MD, General Surgeon, Hospital Universitário Walter Cantídio, UFC, Fortaleza-CE, Brazil. Technical procedures, critical revision.; III PhD, Assistant Professor, Professional Master’s Degree Program in Minimally Invasive Technology and Simulation in Health, Centro Universitário Christus (UNICHRISTUS), Fortaleza-CE, Brazil. Conception and design of the study, statistical analysis, interpretation of data, critical revision.; IV PhD, Hospital Geral Dr. César Cals, Professional Master’s Degree Program in Minimally Invasive Technology and Simulation in Health, UNICHRISTUS, Fortaleza-CE, Brazil. Conception and design of the study, critical revision.; V PhD, Professional Master’s Degree Program in Minimally Invasive Technology and Simulation in Health, UNICHRISTUS, Fortaleza-CE, Brazil. Conception and design of the study, interpretation and analysis of data, critical revision, final approval.; VI MD, Holy House of Mercy of Fortaleza, Professional Master’s Degree Program in Minimally Invasive Technology and Simulation in Health, UNICHRISTUS, Fortaleza-CE, Brazil. Conception and design of the study, interpretation and analysis of data, critical revision, final approval.

**Keywords:** Education, Medical, Simulation Training, Anastomosis, Surgical, Laparoscopy, Checklist

## Abstract

**Purpose:**

To create a checklist to evaluate the performance and systematize the gastroenterostomy simulated training.

**Methods:**

Experimental longitudinal study of a quantitative character. The sample consisted of twelve general surgery residents. The training was divided into 5 sessions and consisted of participation in 20 gastroenterostomys in synthetic organs. The training was accompanied by an experienced surgeon who was responsible for the *feedback* and the anastomoses evaluation. The anastomoses evaluated were the first, fourth, sixth, eighth and tenth. A 10 item checklist and the time to evaluate performance were used.

**Results:**

Residents showed a reduction in operative time and evolution in the surgical technique statistically significant (p<0.01). The correlation index of 0.545 and 0,295 showed a high linear correlation between time variables and Checklist. The average Checklist score went from 6.8 to 9 points.

**Conclusion:**

The proposed checklist can be used to evaluate the performance and systematization of a simulated training aimed at configuring a gastroenterostomy.

## Introduction

 It is compulsory to define a training program for the teaching of laparoscopic surgery^1^ through the simulators use and a structured curriculum^2^. The simulated training of a manual gastrointestinal anastomosis can be used to teach the skills needed to perform complex procedures via the laparoscopic method^3^.

 The surgical simulation needs to identify a goal, systematize a training, use performance evaluation tools and perform evaluations with the purpose of validating the effectiveness of the proposed educational program^4^. Twenty-four specialists in surgical education in several countries suggest that a curriculum based on the simulated training of any surgical procedure should create, evaluate and implement a specific assessment tool for the exercise performed^5^. An interview with surgeons and residents of General Surgery of three different services came to the conclusion that technical evaluation is essential for quality training in laparoscopic surgery^6^.

 An evaluation tool creation contributes to the technical performance monitoring in an objective and trustworthy manner^7^. A well-structured checklist showed to be able to analyze progress in the ability to make a vascular anastomosis^8^. There is a need to develop an assessment tool to be inserted into a training curriculum for a gastroenterostomy. Therefore, the purpose of this study is to create a Checklist to evaluate performance and systematize the simulated training of a gastroenterostomy.

## Methods 

 This longitudinal experimental study was approved by the Research Ethics Committee of Centro Universitário Christus (number 1,317,965) and Brazil platform system (Approval with CAAE number 49573215.7.0000.5049). 

 This study respects the ethical precepts of human research and presents no possibility of damage to the physical, biological, psychic, moral, intellectual, social, cultural or spiritual dimension of the human being, at any stage of research or as a result of it. The research was carried out in the Laboratory of Surgical Skills of Centro Universitário Christus, located in the city of Fortaleza, Ceará-Brazil. Twelve residents of General Surgery at the end of the second year of training participated in the study.

### Surgical procedure

 After a first theoretical session consisting of a basic course of endosutures, videos and orientations, the residents began the training which aimed at the participation in the making of twenty anastomoses, being ten as main surgeon and ten as assistant surgeon. Dual training was used to strengthen teamwork and contribute to learning through error analysis. The complexity of the procedure, too, requires a helper. The organs used during training with the residents were a stomach and a segment of synthetic jejunum. The gastroenterostomy was carried out through a continuous suture in single plane with two Seda 3.0 wires^9^. 

 The procedures were distributed in five sessions, with approximate interval of one week and total duration of six weeks. The training was accompanied by an experienced surgeon who was responsible for the feedback and evaluation of anastomoses. The anastomoses evaluated were the first, fourth, sixth, eighth and tenth. The first one chosen to serve as the initial evaluation. In the other sessions the last anastomosis was evaluated. The simulation was performed in the Endosuture Trainning Box. 

### Evaluation of anastomoses

 The evaluation of the anastomoses was done by the same surgeon and occurred during the training. The evaluator was previously trained to evaluate the participants uniformly. A 10-item Checklist (Chart 1) was used which was elaborated by three surgeons with experience in simulation of surgical procedures and anastomoses by laparoscopy in real patients. The time to perform the procedure, too, was noted. The timing began with the wire entry into the simulator cavity and ended with the removal of the wire.

**Chart 1:** Checklist for laparoscopic gastroenterostomy training.

Questions evaluated	Incorrect	Correct
Fitted firm knots and external to the anastomosis (First double and two simple ones).		
Needle positioning on the Needle Holder (1 \ 3 distal with 90 degree angle).		
Needle penetration (90° skin tissue inlet making the curvature at the exit, with smooth movements and without damaging the tissue).		
Use both hands in a coordinated way (Skin tissue presentation and needle assembly).		
Skin tissue amount (Penetrate the needle in the same skin tissue amount on both sides of the anastomosis, avoiding picking up too much or too little).		
Handling the surgical thread (It draws the thread in its most proximal portion to the tissue. It does not break or damage the thread and removes its remains from the simulator).		
Equivalent distance between points (4 to 6 mm, leaving no redundancy between the angles).		
It uses the wizard (tissue exposure, suture pull and camera manipulation).		
Operation flow (Starts with posterior anastomosis and ends with anterior anastomosis using appropriate tweezers and 2 wires).		
Persistent and intact anastomosis (Diameter greater than 3cm and absence of visible fenestrations).		
**Total of points:**		

### Statistical analysis

 Quantitative numerical results were presented as measures of central tendency. Normality tests were performed for numerical variables. Depending on the normality of the variables, ANOVA or Mann-Whitney tests were performed, as appropriate. Simple linear regression and multiple analyzes were performed to verify the statistical significance of the correlations. Comparisons with p value up to 0.05 were considered significant. The data were tabulated and analyzed by the SPSS software (Statistical Package for the Social Sciences), v23, SPSS, Inc. for the analysis and evaluation of the collected data.

## Results

**Figure 1:**
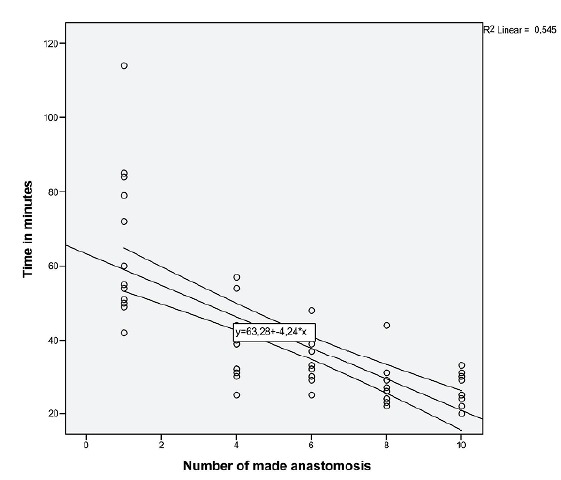
Relationship between the number of anastomoses made and time.

Figure 1 shows a significant reduction in operative time to make a gastroenterostomy. The correlation index (r) of 0.545 represents a high linear correlation between the variables, and the p <0.01 a statistically significant result.

**Figure 2:**
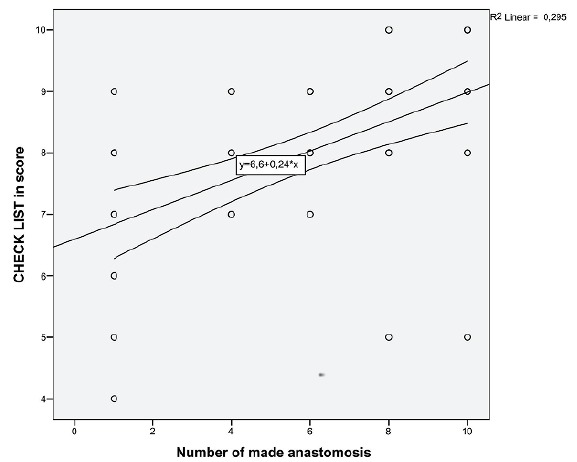
Relationship between the number of anastomoses made and the score in the Checklist.

Figure 2 shows an improvement in the Checklist score during the training of gastrojejunal anastomoses. The average score went from 6.8 to 9 points. The correlation index (r) of 0.295 shows a high linear correlation between the variables, and p <0.01 a statistically significant result. Thus, there was an improvement in the quality of the anastomoses and the operative technique at the training end.

## Discussion

 The technical ability evaluation through different evaluation instruments is useful in teaching the surgery, since it can define a goal to be obtained. Currently, there are several ways to analyze proficiency in performing surgical procedures. However, new evaluation tools need to be developed to analyze the capacity to perform increasingly specific tasks^10^. Some skills assessment methods are: observation by specialists using global assessment scales and specific checklist for a given task, computer video analysis and mechanical outcome metrics (eg, anastomoses mechanical integrity)^11^.

 The global assessment scale Objective Structured Assessment Technical Skills (OSATS) is applied to any assessment of surgical skills and assesses knowledge, manipulation skill, and action record. It consists of seven assessment items on a 5-point Likert scale. The minimum score of each participant may be 7 points and the maximum of 35 points, having to reach 21 points or more to be considered competent in an individual task^12^ ^,^ ^13^. The OSATS scale with some modifications was used to evaluate the evolution of twenty-four general surgery residents during a training in five different practice stations. At the training end, it was observed that this scale can establish a learning curve and thus allow adequate surgical skills progression monitoring^14^.

 The OSATS scale has a specific checklist for suture and can be a useful tool for evaluation and teaching different techniques of laparoscopic sutures and intracorporeal skills^15^. A simulated training of ten gastroenterostomy shows a statistically significant improvement (p<0.01) in the OSATS scale score. The evolution of the surgical technique and the final quality of the anastomosis was important, resulting in the last operation an average score of 33.4 points on the OSATS scale. There is a high linear correlation between the improvement in the OSATS scale score and the number of anastomoses made^9^.

 A short training for forty-eight general surgery residents used the OSATS scale and a structured Checklist to evaluate the making of an intestinal anastomosis. There was a significant improvement in the score of both assessment tools after training. Some Checklist items were: the thread and needle proper selection, needle and tissues manipulation, spacing between points (3 to 5 mm), similar tissue amount on both sides of the suture, three adjusted nodes and operation flow^16^. At the end of the general surgery residency, six residents were evaluated concerning the ability to perform an intestinal anastomosis and a high number of errors were observed despite a high score on the OSATS scale. Therefore, it is important to use more than one evaluation tool to improve awareness of the need for additional learning^17^.

 Global Operative Assessment of Laparoscopic Skills (GOALS) is easy to use, reliable, valid and can be an effective means of providing surgeons in training feedback on the skills development in laparoscopic surgery. There are 5 items that score from 1 (lowest level of performance) to 5 (best performance level) and can vary the final score between 5 and 25. The evaluated items are related to depth perception, bimanual dexterity, efficiency, tissue manipulation and autonomy. The items are specific to evaluate laparoscopic skills, but do not evaluate a specific procedure^18^.

 A training conducted by 32 volunteers (surgeons, residents and medical students) showed that the GOALS is suitable for performance evaluation in laparoscopic surgery after using the MacGill Inanimate System for Training and Evaluation of Laparoscopic Skills (MISTELS)^19^. The MISTELS exercises Comprise five tasks inspired by typical tasks performed during laparoscopic cholecystectomies, appendicectomies, inguinal hernia repair or Nissen fundoplication. The tasks are pegboard transfers, pattern cutting, a ligating loop placement, extracorporeal and intracorporeal knot^20^. The MISTELS system has been further validated since the original study and has been shown to be highly reliable and valid system^20^ ^,^ ^21^.

 A specific checklist for anastomoses training may contribute to performance evaluation^16^. Some important items that should be included in the Checklist of a particular surgery can be obtained while viewing common technical errors of expert surgeons and beginners. These are important items that should be included in a Checklist to evaluate a suture: needle positioning and conduction, tissue handling and damage, similar distance between stitches, continuous suture flow, wire manipulation, surgical nodes quality, and the use of both hands^22^.

 A laparoscopic stitch training and surgical nodes used a Checklist to evaluate the proficiency acquisition. The checklist was composed by the following items: distance between knots (5 to 7 mm), Tissue margins (4 to 5 mm), Symmetry of the edges of knots and Adequate Knot tension. Another variable analyzed was the knots amount in 18 minutes. This teaching model was able to show the evolution in the learning curve^23^.

 Technical competence can be assessed by creating specialist scales that are based on the degree of support that residents need during a particular stage of the surgical procedure. For example, a score of 1 can be established for the need for total support of the expert surgeon, score 5 when there is safety in performing the procedure without any kind of help, and score 6 for the cases where the procedure was performed perfectly^24^.

 Brazilian surgeons have created a questionnaire to evaluate the learning of general surgery residents during their training. Four questions were asked for eleven different types of operations. The questions were related to knowledge of the anatomy, operative technique and surgical ability^25^. A questionnaire with several items related to a training curriculum can be used to evaluate satisfaction with a specific training program^26^.

 A study analyzed a suture training in ex vivo pig’s stomach and registered through Motion analysis metrics the evolution in the development of the proposed task. The time of execution and the length of the path traveled by laparoscopic instruments should be used in the evaluation of movements in a laparoscopic suture exercise. Therefore, the analysis of movements is effective as a method for objective evaluation of psychomotor skills in laparoscopic suture. However, this method does not take into account the quality of the suture^27^. An effective method for assessing the progression of psychomotor skills during the manufacture of an intestinal anastomosis employed internal measurements of air pressure and image processing during the training of 53 surgeons. In the analysis of the prepared anastomosis, the following criteria were used: volumes of air pressure leak, numbers of full-thickness sutures, suture tensions, areas of wound-opening and performance times. The system used seems to be useful in assessing the progression of skills in laparoscopic suture^28^.

 A simulated training program in laparoscopic anastomosis selected twelve residents of surgical specialties who attended 4 weeks during one year (20 hours per week) to perform the proposed task. There was an average of 15.8 enteroanastomoses per resident and 16.4 gastroenteroanastomoses per resident. The time to perform the anastomoses reduced as time passed by and reached the plateau after 70 hours of training. Some of the criteria used to evaluate the quality of anastomoses were: anastomosis leak test after hydrostatic test with saline solution and adequate suture tension. There was a lack of an evaluation tool to systematize the exercise and to evaluate in more detail the evolution of the technique and quality of the anastomosis^29^.

 Some tips to assist the evaluation of surgical performance are: choosing the right evaluation tools, using generic and specific evaluation scales, observation during training should be prioritized, avoiding evaluator bias, evaluator training, using the most evaluations and possible advisers, prioritize formative evaluation in relation to summative, and finally, monitor and report the experiences and results^30^.

 In controlled environments the variance across predictive measurements is likely to be low, and therefore R2 values can be expected to lie in the 0.8 range. In clinical studies, however, R2 values vary widely depending on the nature of the analysis. For example, when comparing associating surgical technical factors, values of R2 are reported in the 0.2 to 0.4 range^31^. It was evident that during the training the time for making anastomoses and the increase in Checklist scores had a high correlation index and a statistically significant result. It is important to introduce this assessment tool into a structured training curriculum. Simulated training of a gastroenterostomy can be performed with good results when using a black box, silk threads, good quality tweezers and synthetic organs^32^.

## Conclusion

 The proposed Checklist can be used to evaluate the performance and systematization of simulated training aimed at making a gastrostomy.
